# Computational Approaches to Toll-Like Receptor 4 Modulation

**DOI:** 10.3390/molecules21080994

**Published:** 2016-07-30

**Authors:** Jean-Marc Billod, Alessandra Lacetera, Joan Guzmán-Caldentey, Sonsoles Martín-Santamaría

**Affiliations:** Department of Chemical & Physical Biology, Centro de Investigaciones Biológicas, CIB-CSIC, C/Ramiro de Maeztu 9, 28040 Madrid, Spain; jmbillod@cib.csic.es (J.-M.B.); a.lacetera@cib.csic.es (A.L.); juanguzman@cib.csic.es (J.G.-C.)

**Keywords:** Toll-like receptor 4, TLR4/MD-2 modulators, molecular recognition, drug design, computational chemistry, MD simulations, docking, homology modeling, virtual screening

## Abstract

Toll-like receptor 4 (TLR4), along with its accessory protein myeloid differentiation factor 2 (MD-2), builds a heterodimeric complex that specifically recognizes lipopolysaccharides (LPS), which are present on the cell wall of Gram-negative bacteria, activating the innate immune response. Some TLR4 modulators are undergoing preclinical and clinical evaluation for the treatment of sepsis, inflammatory diseases, cancer and rheumatoid arthritis. Since the relatively recent elucidation of the X-ray crystallographic structure of the extracellular domain of TLR4, research around this fascinating receptor has risen to a new level, and thus, new perspectives have been opened. In particular, diverse computational techniques have been applied to decipher some of the basis at the atomic level regarding the mechanism of functioning and the ligand recognition processes involving the TLR4/MD-2 system at the atomic level. This review summarizes the reported molecular modeling and computational studies that have recently provided insights into the mechanism regulating the activation/inactivation of the TLR4/MD-2 system receptor and the key interactions modulating the molecular recognition process by agonist and antagonist ligands. These studies have contributed to the design and the discovery of novel small molecules with promising activity as TLR4 modulators.

## 1. Introduction

Toll-like receptors (TLRs) are pattern recognition receptors with a primordial role in the activation of the innate immunity, and in particular, the Toll-like receptor 4 (TLR4) is the mammalian endotoxin sensor [1]. TLRs are trans-membrane proteins located extra- and intra-cellularly and specialized in the recognition of pathogen-associated molecular patterns (PAMPs) [2]. Among the different TLRs, TLR4 represents an interesting case of study for several reasons: (i) it is the only TLR that needs an accessory protein (myeloid differentiation factor 2, MD-2) to become active; (ii) it can activate the immune response through two different signaling pathways; and (iii) when faced with the specific PAMP lipopolysaccharides (LPS), a component of the outer membrane of Gram-negative bacteria, TLR4 can recognize a wide variety of agonist and antagonist compounds with different chemical structures [3].

**Figure 1:**
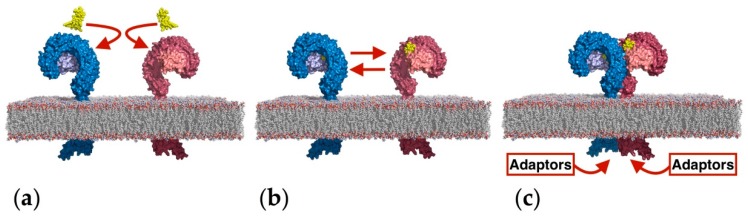
Schematic representation of the LPS-induced dimerization of the TLR4/MD-2 complex leading to immune system activation. Red arrows indicate motion and mutual recognition. (**a**) Two LPS (yellow) are engaged by two distinct TLR4/MD-2 systems (blue and red, MD-2 in pale blue and pale red); (**b**) two TLR4/MD-2/LPS complexes dimerize by protein-protein interactions at the dimerization interface; (**c**) dimerization brings together the two intracellular TIR-containing domains providing a suitable molecular surface for recruiting downstream adaptors.

The immune response mediated by the activation of TLR4 and its co-receptor MD-2 [4] starts with the collection of an LPS by the transport protein cluster of differentiation 14 (CD14), which delivers it MD-2 (Figure 1). The eventual formation of the TLR4/MD-2/TLR4*/MD-2* heterodimer promotes the dimerization of the intracellular domains, which finally leads to the recruitment of downstream adaptors and to the activation of the intracellular signaling events and final immune response. Thus, the binding of an antagonist ligand to the extracellular domain would prevent the formation of the dimer, and consequently, the intracellular signaling events would not occur.

**Figure 2:**
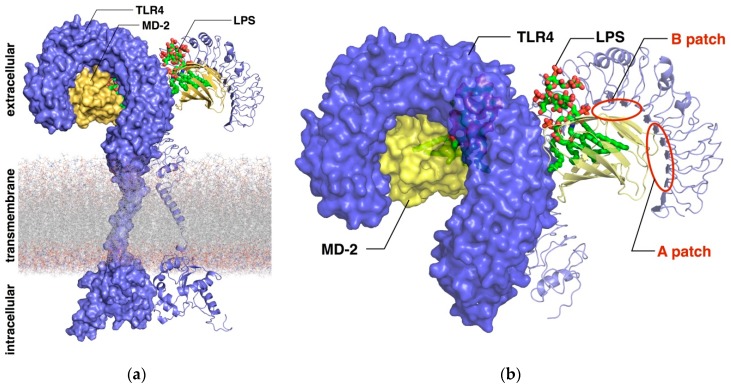
Representation of the 3D structure of TLR4/MD-2/LPS. (**a**) Large-scale representation showing the intracellular, transmembrane and extracellular domains of TLR4/MD-2 in complex with *E. coli* LPS. 3D Structures correspond to the X-ray crystallographic structure for the extracellular domain (PDB ID 3FXI) and homology modeling for the transmembrane and intracellular domains. (**b**) Close-up look at the TLR4 extracellular domain (purple) along with MD-2 (yellow) and LPS (CPK colors with C atoms in green) from PDB ID 3FXI.

**Figure 3:**
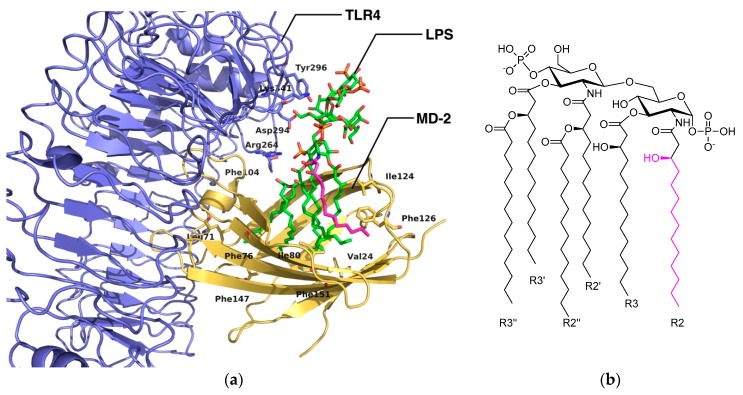
Representation of the LPS in complex with TLR4/MD-2. (**a**) Detail of the 3D structure of the complex between TLR4/MD-2 and *E. coli* LPS (CPK colors with C atoms in green and R2 C atoms in magenta) from the X-ray crystallographic structure (PDB ID 3FXI); (**b**) chemical structure of *E. coli* lipid A. The R2 FA chain (magenta) placed at the channel of MD-2 completes the dimerization interface.

**Figure 4:**
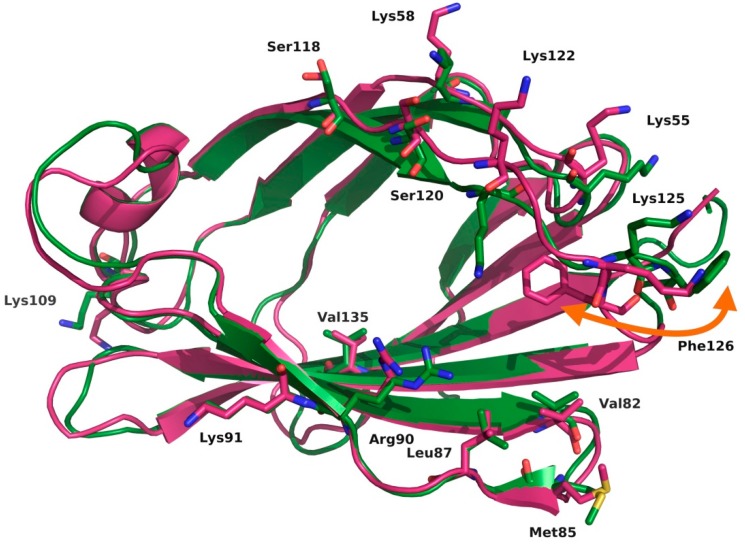
Superimposition of the X-ray crystallographic structures of the agonist (magenta) and the antagonist (green) conformations of MD-2 from, respectively, PDB ID 3FXI and 2E56. Bound ligands have been hidden for the sake of clarity (*E. coli* LPS in 3FXI; three myristic acids in 2E56). Conformational change of the molecular switch Phe126 is marked.

LPSs are large glycolipids consisting of a highly conserved lipid moiety, known as lipid A, and a polysaccharide composed of a core oligosaccharide (outer and inner part) and a *O*-specific polysaccharide (Figure 2 and Figure 3 and Figure S1 in the Supplementary Materials). In recent years, structural information on TLR4 has been available, providing insights on the binding at the atomic level [3]. In the X-ray crystallographic structure of TLR4/MD-2 in complex with potent agonist *Escherichia coli* LPS (PDB ID 3FXI), MD-2 is able to accommodate up to five fatty acid (FA) chains of the *E. coli* lipid A into its large hydrophobic cavity. The sixth chain (FA chain R2 in pink; see Figure 3b) protrudes from the MD-2 hydrophobic pocket, thus completing the dimerization interface. The phosphate groups of the LPS are anchored to the polar MD-2 rim (e.g., Arg90, Lys91, Ser118 and Lys122; Figure 4), and the polysaccharide moiety establishes a network of polar interactions with TLR4. Two distinct protein-protein interaction regions have been defined between TLR4 and MD-2, the so-called patches A and B (Figure 2b and Figure S1), which respectively contain residues close to the N-terminal, and the central domain of TLR4. Therefore, all of the structural components of the LPS molecule play a fundamental role in the TLR4/MD-2 recognition and in the binding [5].

Lipid A is composed of FA chains of different lengths attached to a 1,4-β-diphosphorylated diglucosamine backbone [6]. The agonistic activity of lipid A has been mainly assigned to the number (established as six), length and chemical structure of the attached FA chains, as well as variability in the level of phosphorylation and the number and types of substituted groups found attached to the phosphate residues [6]. Recent findings have pointed to the need of revisiting this paradigm since some examples of LPS bearing a penta-acylated lipid A, together with positively-charged residues decorating the lipid A, have been reported with immunostimulatory ability [7,8]. Therefore, these data suggest that subtle changes in lipid A structure may profoundly impact the innate immune response from the host.

Regarding the recognition by MD-2, the placement of the sixth FA chain into one particular hydrophobic channel of MD-2 contributes to complete the dimerization surface, thus allowing the binding with the partner TLR4* (Figure 3) [9,10]. The assumption is that Phe126 is the “molecular switch” in endotoxic signaling. Upon lipid A binding, the flexible MD-2 protein experiences a local conformational change involving the Phe126 side chain and the surrounding residues (the loop formed by residues 123–129) (Figure 4) [11].

Interestingly, tetraacylated lipid IVa, a lipid A precursor, binds in an antagonistic manner to human MD-2, whereas it binds in agonist manner in mouse MD-2 [12]. This causes a proinflammatory effect in mouse cells (agonism), but it remains inactive in human. This behavior was also studied in other mammalian species. For example, lipid IVa seems to be a weak agonist in equine cells, while it antagonizes the lipid A-induced activation of canine TLR4/MD-2. From the X-ray crystallographic structures of *h*MD-2/lipid Iva (PDB ID 2E59) and *m*MD-2/lipid IVa (PDB ID 3VQ1) complexes, it was observed that the four FA chains of lipid IVa are inserted into the MD-2 pocket, occupying a similar volume in the case of both human and mouse TLR4/MD-2, although with different consequences in the agonist (human)/antagonist (mouse) activity. Importantly, from the crystal structures, it has been elucidated that the orientation of the lipid IVa is rotated by 180°, i.e., lipid IVa presents two different molecular patterns for the distinct human vs. mouse interaction [11,13,14]. These different binding modes of lipid IVa, mainly determining how the phosphate groups interact with the TLR4, may be crucial for its distinct behavior in different species. It is therefore evident that the key ligand/receptor interactions must be scrutinized in order to rationalize the mechanism for TLR4/MD-2 modulation.

Elucidation of the molecular determinants for a molecule to be an agonist or an antagonist of TLR4, together with the understanding of the mechanism of the TLR4/MD-2 system, has high potential for the design of new molecules able to modulate TLR4 immune response. In this context, computational studies can be of great help. TLR4 has attracted much attention for the finding of new modulators with important applications in biomedicine [3,15], and several new compounds modulating TLR4 are undergoing preclinical and clinical evaluation, for the treatment of sepsis, inflammatory diseases and rheumatoid arthritis, as well as vaccines and cancer immunotherapeutics [16].

Given the complexity of the TLR4/MD-2 system, its study represents, with no doubt, a challenge for computational biological chemists. This review presents the recent contributions that have given interesting insights into the mechanism behind the TLR4/MD-2 activation/inactivation and into the molecular recognition process at the atomic level. These studies are paving the way for future developments in this field, such as the design and discovery of novel small molecules with promising activity as TLR4 modulators. Moreover, the computational studies reviewed here represent cutting-edge examples of the applications of computational techniques to the study of complex biomolecular systems.

## 2. Computational Studies of the TLR4/MD-2 Ectodomain

There are several studies in the literature focused on the extracellular domain of the TLR4/MD-2 complex, more specifically on its ability to recognize lipid A and lipid IVa. Garate et al. [17] report MD simulations of at least 11 ns of apo-MD-2, TLR4/MD-2 dimer, MD-2/lipid A, MD-2/lipid IVa and TLR4/MD-2/lipid A complexes; they conclude by highlighting the hydrophobicity of the MD-2 pocket and its ability to close promptly in an aqueous environment. According to the authors, the flexibility of the helix connecting MD-2 with TLR4 (helix H1) is essential for the observation of this behavior, and they propose a putative equilibrium between the open and the closed states of MD-2. MD-2 has been observed to close at a similar rate in the simulations where it was in the presence or in the absence of TLR4. However, in the former case, MD-2 was observed to fluctuate less due to the presence of TLR4, reducing the number of degrees of freedom. Another interesting conclusion by the authors is the key role that charged phosphates play in the early recognition of lipids with the corresponding impact on the formation of heterotetramers. The MD simulations performed on the TLR4/MD-2/lipid A complex also showed that the presence of the ligand energetically stabilizes the complex, indicating cooperativity in the binding process. Evidence of the plasticity of MD-2 has also been observed by DeMarco et al. after several MD simulations performed in complex with variably-acylated lipid A molecules from *Escherichia coli* and *Neisseria meningitidis*. The results of these simulations (50 ns for each production run) led to the conclusion that the level of acylation of these ligands greatly influences the final architecture of the dimerization interface [18], leading to agonist or antagonist conformation of the TLR4/MD-2 system. Paramo et al. performed long MD simulations of at least 100 ns on the TLR4/MD-2 system in complex with different ligands observing the Phe126 transition from a closed (agonist/active conformation) to an open (antagonist/inactive conformation) state in the presence of lipid IVa, Eritoran and in the apo-form [19]. The dimerization interface between the two partners’ heterodimers (TLR4/MD-2/TLR4*/MD-2*) was destabilized in agonist-free systems, especially due to the opening of the Phe126 switch, which disrupts the arrangement of nearby side chains from Leu87, Val82 and Met85 of MD-2. These simulations are in agreement with the NMR studies pointing at the re-orientation of the Phe126 aromatic side chain induced by the binding of hexa-acylated endotoxin [20]. As can be observed, the lengths of the MD simulations range from relatively short to longer ones, depending on the origin (experimental vs. homology modeling) of the starting geometries, as well as the aim that is pursued: geometry optimization, study of the stability of the ligand-protein/protein-protein interactions, flexibility studies, binding free energy calculations, etc.

### 2.1. Computational Perspectives of the TLR4/MD-2/Ligand Recognition Process and Dimerization Event

The TLR4 ectodomain and its dimerization mechanism have been the main subject of several computational studies. MD simulations have been reported by de Aguiar et al. of TLR4 alone, MD-2 alone, TLR4/MD-2 complex and TLR4/MD-2/TLR4*/MD-2* complex [21]. The simulations of the TLR4 ectodomain revealed pronounced conformation and structure alterations in the N- and C-terminal domains, showing higher RMSD values compared to the full protein RMSD values. Furthermore, after 100-ns MD simulation, the distance between *N*-terminal and *C*-terminal regions increased from 5.7 Å–10.9 Å, suggesting an opening of the TLR4 curvature. When the MD simulations were performed in the TLR4/MD-2, these fluctuations became attenuated, indicating a stabilizing role of the MD-2 protein. MD simulations on the MD-2 protein alone showed high mobility of the loops, especially the one containing Lys109 and the region comprising residues Lys55 and Lys58. Interestingly, the Lys55-Lys58 region does not interact directly with TLR4, as is observed in the crystal structure of the TLR4/MD-2 complex (PDB ID 3FXI). However, throughout the MD simulation of the TLR4/MD-2/TLR4*/MD-2* complex, MD-2 underwent structural rearrangements and interacted with TLR4 and partner TLR4*, reinforcing the stabilizing role of MD-2 for the TLR4 complexation.

**Figure 5:**
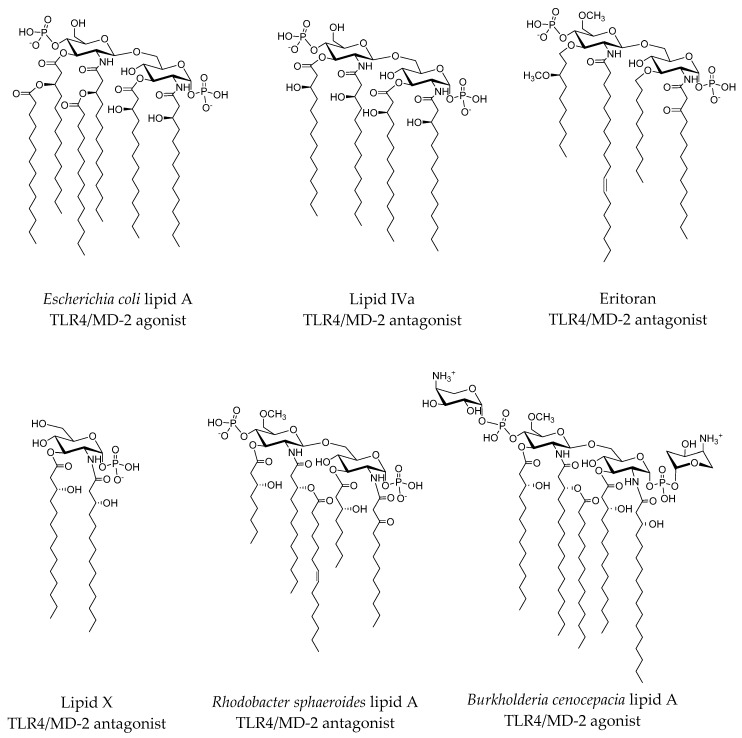
Lipid A and synthetic lipid A analogues with activity as TLR4 modulators. Activity is referred to *h*TMR4/MD-2.

In a related work, Anwar et al. performed interesting computational studies of the TLR4 signaling mechanism by studying the species-specific behavior of TLR4/MD-2 in the recognition of *Rhodobacter sphaeroides* lipid A (RsLA; see Figure 5) [22]. The penta-acyl chain-containing RsLA is midway between of the agonist (six FA chains) and antagonist (four FA chains) structures; it activates the TLR4 pathway in horses and hamsters, while inhibiting it in humans and mice, raising interesting questions about the molecular recognition process. The authors docked RsLA on the four human, murine, horse and hamster TLR4/MD-2 systems and performed 25-ns MD simulations of the docked complexes, revealing several insights regarding the RsLA molecular recognition event by TLR4/MD-2. Over the simulation time, local and global mobility, the surface accessible solvent area (SASA) of the ligand and the surface charge distributions of TLR4 and MD-2 were monitored. The GlcN1-GlcN2 backbone was shown to acquire agonist-like conformation in horse and hamster TLR4/MD-2 and antagonist-like conformation in the human and murine systems. Evaluation of the stability of the on/off switch Phe126 loop of MD-2 (residues 123–129) showed that the human and murine loop was less stable than horse and hamster loops. Moreover, the RMSD of the MD-2 loop containing residues 81–89, which are the residues interacting with the partner TLR4*, thus mediating the dimerization event, showed a greater variation in humans and mice, in comparison to the horse and hamster systems. These data suggest a relationship between the flexibility of both loops (residues 81–89 and residues 123–129) and the agonist/antagonist activity of the ligand and provide a plausible explanation for the species-specific behavior of RsLA regarding TLR4 activation.

### 2.2. Computational Strategies to Study Mutant TLR4 and MD-2 Proteins

Herein, we report three studies that used in silico-mutated TLR4/MD-2 systems to serve different purposes; namely, to transform the mouse complex interface into the human one by mutating the residues laying there into their human counterparts, to estimate the influence of the mutations on the binding affinity of a ligand and to evaluate the impact of mutations on the structural shape and plasticity of the MD-2 binding pocket.

In a 2009 study, Slivka et al. [23] used Rosetta software [24] to compare the binding energy of a truncated MD-2 with the original one. MD-2 was truncated (termed MD-2-I) to keep only the residues identified as playing a major role in maintaining the TLR4/MD-2 heterodimer stability. The docking experiment was performed targeting both a partial human TLR4 retrieved from the Protein Data Bank (PDB ID 2Z65) and a full-length TLR4 humanized model built by mutating the residues at the TLR4/MD-2 heterodimer interface in the mouse crystal structure (PDB ID 2Z64) into their human counterparts (TLR4: F160L, G234N, K263R, D264N, T290A; MD-2: H96R, H98R). In the first case, the affinity of MD-2-I was found higher than the one of the full-length MD-2. When docked against the human TLR4 model, MD-2-I exhibited a lower affinity than the full length MD-2. Altogether, these results indicate that MD-2-I is theoretically able to bind TLR4 and might even compete with the full-length MD-2. This was confirmed by cell assay experiments showing that the addition of compound MD-2-I abolishes their responsiveness to LPS stimulation. Flow cytometry analyses on cells (HEK293 cell line, transfected with all proteins involved in the TLR4 activation pathway) incubated with LPS covalently linked to fluorescein isothiocyanate (LPS-FITC), suggest that MD-2-I impedes TLR4/MD-2 dimerization. The SEAP assay shows that MD-2-I also alters downstream signaling.

A 4-aminoarabinose-containing lipid A from the opportunistic bacterium *Burkholderia cenocepacia* (Figure 5) and its aminoarabinose-deficient equivalent were docked to *h*MD-2 and *m*MD-2 [7]. These docked models were used to build a full dimer complex in order to perform 0.1-ps MD simulations. In both the human and the murine systems, the wild-type (WT) LPS obtained a better predicted free energy of binding than the aminoarabinose-deprived one, with both the AutoDock [25] and AutoDock VINA [26] docking programs. An energy analysis was conducted to estimate the per residue contribution to the total ligand binding energy for both WT and mutated TLR4/MD-2/TLR4*/MD-2* complexes (D294A, R322A, S415A* and S416A*) using the MM-ISMSA method [27]. This study permitted the identification of the mutated residues as major contributors to the total binding energy of *B. cenocepacia* LPS and suggested that the ammonium groups of Ara4N stabilize the complex by providing additional anchorage interactions.

Recently, the critical role of residue 135 of MD-2, located deeply inside the hydrophobic pocket, was reported by Vasl et al. [28]. *h*MD-2 has the ability to bind LPS in the absence of TLR4, while *m*MD-2 is responsive to LPS only when engaged in a complex with TLR4. Site-directed mutagenesis was used on *h*MD-2, to mutate Val135 to its murine alanine counterpart. This single point mutation led to a mutant V135A *h*MD-2 lacking the ability of binding LPS. A series of 50-ns MD simulations of the WT MD-2 and the V135A mutant MD-2 in solution and in complex with TLR4 was performed to study the conformational changes. In the case of the WT *h*MD-2, the authors reported an abrupt decreased of the SASA and volume in the first nanoseconds of the simulation, describing it as a hydrophobic collapse. This phenomenon was not observed in the V135A systems, suggesting that Val135 is primordial to confer plasticity to MD-2. This tendency was confirmed by another simulation of MD-2 in complex with three myristic acids (as observed in some crystal structures). The V135A mutant *h*MD-2 needed a much longer simulation time to adapt its shape to the three myristic acids than the wild-type. The authors concluded that this loss of plasticity could incapacitate *h*MD-2 for binding LPS [28].

### 2.3. The Binding Mode of TLR4 Modulators: Computational Approaches

Several computational studies have been performed in order to clarify the binding mode of TLR4/MD-2 agonist and antagonist ligands. As introduced above, the unveiling of the molecular recognition process at atomic detail is one of the major challenges in TLR4/MD-2 modulation. Molecular modeling, docking studies and MD simulations have already provided relevant contributions about the ligand/receptor interactions with promising impact for rational drug design. Relevant residues discussed in this review are displayed in Figures S2 and S3 in the Supplementary Materials.

#### 2.3.1. Synthetic LPS Mimetics

Inspired by the LPS structure, different ligands have been designed and synthesized. One of the first compounds to enter into clinical trials was Eritoran, a synthetic lipid A mimetic, potent TLR4 antagonist, which reached phase III clinical trials as an antisepsis agent, but failed since the study did not meet its primary endpoint of reduction in 28-day all-cause mortality in patients with severe sepsis [29]. Eritoran is a tetraacylated lipid A, the structural analogue of lipid A from RsLA, antagonist of human TLR4 and agonist of TLR4 from mouse and horse. In order to analyze the species-dependent activity of Eritoran, Scior et al. [14] built homology models by means of the SCWRL4 program [30]. Afterwards, docking of Eritoran was performed with AutoDock in order to determine the characteristics of the agonist/antagonist binding in the TLR4 structures from different species: human, mouse and horse. Some key amino acids were identified as relevant in species-specific binding: Lys58 (that corresponds to Asn in mouse and to Glu in horse), Lys388 (which is a Ser in mouse and a Lys in horse) and Gln436 (which is an Arg in mouse and a Gln in horse). The different pattern of interactions that are presented by these different residues impairs the TLR4-TLR4* bridging role of the ligand, thus preventing the effective dimerization and the agonist activity.

**Figure 6:**
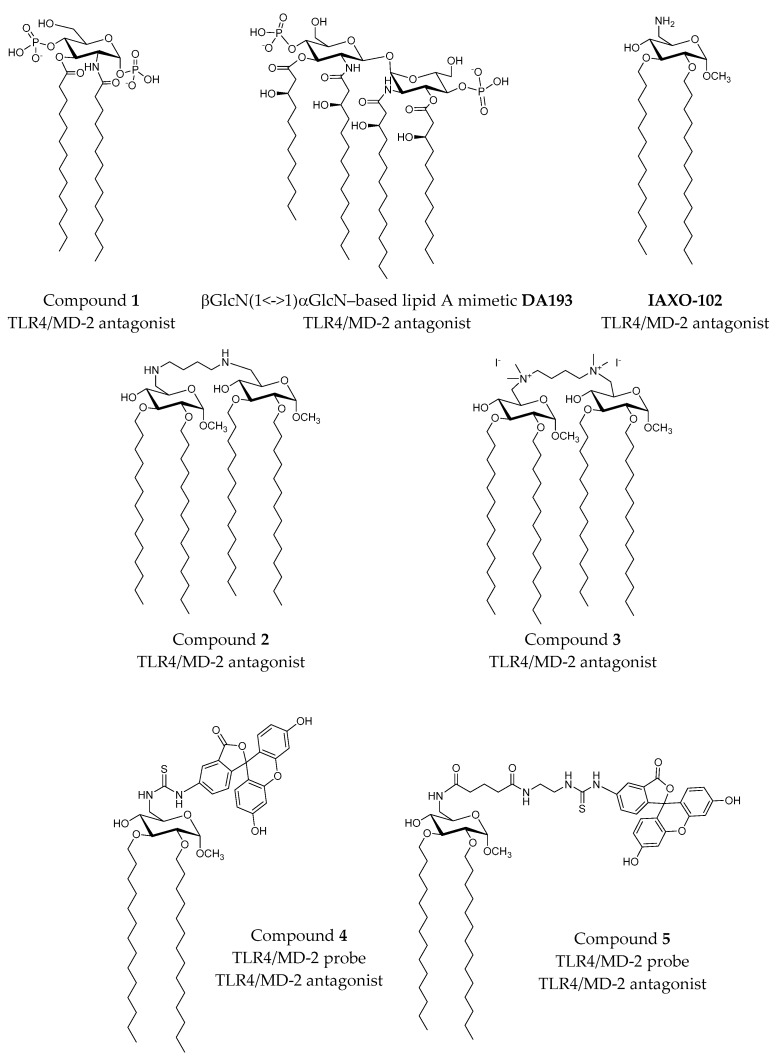
Synthetic LPS mimetics studied by computational approaches.

Modifications of the chemical structure of the lipid A scaffold have served as a starting point for the design of novel TLR4 modulators. One modification reported by Cighetti et al. [31] was the diphosphorylation of the scaffold of lipid X (Figure 5), a biosynthetic precursor of lipid A, leading to Compound **1** (Figure 6), which has been found to be an antagonist in both human and mouse TLR4/MD-2. This compound was also shown to stimulate CD14 internalization in bone-marrow-derived murine macrophages, thus demonstrating targeting of also CD14 in a TLR4-independent manner. In order to propose 3D models for the ligand recognition processes, computational studies were undertaken on both CD14 and MD-2 proteins. Docking calculations in MD-2 with AutoDock and AutoDock VINA [26], followed by MD simulations of the resulting complexes with the Impact program [32], led to the identification of two possible binding poses: the most stable one (in terms of predicted binding energy) allocated both FA chains inside the MD-2 binding pocket, mimicking the lipid IVa binding to MD-2 in the crystal structure (PDB ID 2E59). The MD-2/Compound **1** complex is stabilized by hydrophobic interactions between its two FA chains and aliphatic and aromatic residues from the MD-2 pocket together with polar interactions at the rim of MD-2, involving mainly the phosphate groups and side chains from Ser118 and Arg96 residues and, in some cases, interactions between the amide CO or ester CO groups from Compound **3** and the Ser120 OH group. This result was in agreement with NMR experiments performed by the authors that clearly showed FA chain-protein interactions. In a few cases, calculations predicted a second docked binding pose for Compound **1** presenting only one FA chain inside the MD-2 hydrophobic pocket, while the second FA chain was lying over Ile124. Interestingly, in the agonist conformation, this residue has moved towards the inside of MD-2 and Phe126 occupies its place. This synchronism allows the agonist/antagonist switch. In the bound/unbound equilibrium, this alternative binding pose could co-exist with the first and most stable one. Cighetti et al. [31] also combined docking and MD simulations to propose a binding mode for Compound **1** with CD14. CD14 is also characterized by having a wide lipophilic pocket, but with fewer polar residues at the rim. Compound **1** was predicted to bind with the saccharide moiety and the phosphate groups at the entrance of the CD14 hydrophobic cavity and with the FA chains inside the pocket, in agreement with the CD14 binding properties observed experimentally. In addition to its properties to prevent TLR4 signaling, Compound **1** has also been proposed as a promising hit as TLR4 modulator because of its favorable solubility properties and for its lack of toxicity according to the MTT tests.

Another strategy to mimic lipid A was the design of tetraacylated lipid A mimetics based on the βGlcN (1↔1) αGlcN scaffold analogue by substituting the β(1→6) with βα(1↔1) glycosidic linkage in order to confer rigidity to the molecule [33]. In particular, Compound **DA193** (Figure 6) resulted in being a dose-dependent antagonist in human and mouse, according to assays performed in HEK293 cells transiently transfected with membrane CD14 (*m*CD14)/*h*MD-2TLR4, HEK293 cells transfected with *h*MD-2TLR4 only and assays on human macrophage-like cell line (THP-1). In order to propose an atomistic understanding of the interactions between the ligand and the receptor, MD simulations of 11 ns were performed starting from two possible binding orientations of the ligand into the MD-2 protein: one with the α-GlcN ring facing the Phe126 loop and the second one with the β-GlcN facing the Phe126 loop with an energy difference similar to that found for orientations of lipid A in the binding site of *h*MD-2. Dissociation constants, calculated from MD simulations of the MD-2/DA193 complex, estimated a binding to MD-2 20-fold stronger than lipid A and three-fold more than lipid IVa. It was concluded that the conformational rigidity of the βα(1↔1) diglucosamine backbone of these tetraacylated lipid A mimetics ensures strong binding to MD-2, in two possible binding poses, unlike the native lipid A structures.

The commercial TLR4 antagonist IAXO-102 (Figure 6) [34] has also served as inspiration for the rational design of TLR4 modulators and probes. Recently, the design and synthesis of IAXO-102-based TLR4 modulators were reported by Ciaramelli et al. [35]. The design was based on a previous docked binding mode of IAXO-102 into MD-2 that showed the possibility to host two ligands simultaneously. A dimeric scaffold with two glycolipid units was designed by connecting both units through C4 diamino and di-ammonium linkers (Figure 6; Compounds **2** and **3**). Both compounds were confirmed to inhibit TLR4 activation and signaling in HEK-Blue™ cells expressing *h*TLR4 in a concentration-dependent manner. Unfortunately, these compounds had a very poor solubility in aqueous solution.

The same IAXO-102 scaffold was used to design fluorescent probes (Figure 6; Compounds **4** and **5**) [35]. The fluorescein moiety was chosen as the chromophore, and two thiourea-based linkers with different lengths attached to the C6 position of the glucose moiety were considered. Normal mode analysis of CD14 was used to obtain motions and conformational changes, and docking calculations of both designed probes **4** and **5** were performed in three different conformations. Calculations predicted binding poses in which the fatty acid chains are buried inside the CD14 binding site (human and mouse) with the sugar located in the external portion. The thiourea linker and the fluorescein moiety established polar interactions with the hydrophilic rim without adopting a preferred pose. The best complexes were selected, according to their preferred docked poses, and were submitted to MD simulations and MM-GBSA analysis. In order to perform docking studies in the *h*TLR/MD-2, a hybrid *h*TLR4/MD-2 model in the antagonist conformation was modeled. As expected, predicted binding poses were similar to those found for CD-14, with the FA chains inside the pocket and the fluorescein moiety reaching TLR4 in the case of the longer probe. In fact, calculations of the SASA with CASTP [36] in both CD14 and MD-2 showed that both pockets have similar topologies and volumes. However, the presence of a lower number of polar residues in the rim of CD-14 allows it to recognize a wide range of microbial and cellular molecular determinants, such as lipopeptides to be transferred to TLR2. In contrast, MD-2 rim’s polarity confers selectivity to the protein towards LPS.

#### 2.3.2. Computational Studies of Natural LPSs

*Rhodobacter sphaeroides* lipid A (RsLA, Figure 5) [22] has five acyl chains, with one unsaturated and two shorter chains than *Escherichia coli* lipid A. It is an antagonist in human and mouse, but an agonist in horse, although, intriguingly, the horse TLR4/MD-2 sequence is more closely related to the human sequence than to the mouse one. To clarify the species-specific response, a computational-aided study of the three 3D structures was undertaken. A homology model was built for horse and hamster TLR4/MD-2, with human and murine X-ray crystallographic structures as templates (PDB ID 3FXI and 2Z64) by means of MODELLER [37]. The role of Arg385 had been proven in horse TLR4 complex activation by lipid IVa [38] through polar interactions between the guanidinium moiety and the phosphate group of lipid IVa. In fact, in other species, this residue is substituted by glycine in human and hamster and by an alanine in murine. The docked structure with AutoDock VINA of the horse TLR4/MD-2/RsLa complex closely resembled the pose of lipid IVa in murine crystal structure of TLR4/MD-2. On the contrary, the docked binding pose found in the hamster MD-2 was similar to the lipid IVa pose in the crystal structure from chicken (PDB ID 3MU3) and human. The difference between the species was mainly attributed to the different characteristic of each protein. By docking studies on *h*MD-2 with AutoDock, it has been observed that the longest chain of RsLPS could be accommodated in MD-2 by folding the chains itself as has been observed with the Eritoran fatty acid chains. The polar head (diglucosamine) is always exposed to the solvent.

Molecular modeling by Irvine et al. has also showed that the different human/horse TLR4 responses towards RsLA is related to two different amino acids, Gly384 and Ser441, in human TLR4 (Arg385 and Pro442 in horse) [39]. Residue Arg385 in horse TLR4, although located around a 9 Å distance from the docked RsLA, could establish a long-range electrostatic interaction with a phosphate group of RsLA, while the Pro442 is situated near the dimerization interface with TLR4* and interacts with an FA chain of RsLA by van der Waals interactions. This hypothesis was confirmed by experimental assays with transfected HEK293 cells with G384R/S441P hTLR4 with *eq*MD-2 and R385G/P442S eqTLR4 with *h*MD-2. It was observed that the R385G/P442S mutations in horse caused a complete loss of activity, and in human, the double mutant G384R/P441S TLR4 was unable to activate the signaling event. Since the double mutation did not revert the activity, other residues must be required. The docking of RsLA in human TLR4/MD-2 shares some similarity with the Eritoran crystal structure, such as the folding of the longest acyl chain and the polar interaction with charged residues of MD-2. RsLPS can adopt two orientations depending on the position of 1-PO_4_ (oriented towards primary TLR4 in the case of horse and towards partner TLR4* in the case of human). This fact leads to different contacts between acyl chains of RsLPS and the hydrophobic pocket of MD-2. Moreover, superimposition of docked RsLA with X-ray crystallography poses of lipid A and lipid IVa showed that RsLA and lipid A acyl chains occupy more volume than lipid Iva, an, more importantly, the R2 chain of RsLA and lipid A protrudes from MD-2 (Figure 3) and establishes interactions with the partner TLR4 in contrast to the R2 chain of lipid IVa, which is folded into the MD-2 pocket.

As mentioned above, the severe pathogen *B. cenocepacia* LPS has been reported by Di Lorenzo et al. to strongly activate human TLR4/MD-2, despite the fact that its lipid A has only five acyl chains [27]. The Ara4N residues in lipid A have been shown to contribute to TLR4-lipid A interactions, and experiments in a mouse model of LPS-induced endotoxic shock confirmed the proinflammatory potential of *B. cenocepacia* penta-acylated lipid A. A combination of docking calculations and MD simulations, together with experimental mutagenesis of the TLR4/MD-2 interacting surfaces, suggested that the longer acyl chains allow reaching deeper regions inside the MD-2 pocket, thus compensating the absence of one FA chain and, at the same time, allowing the exposure of the fifth FA chain on the surface of MD-2. This enables interactions with partner TLR4* and promotes its dimerization. The replacement of Val82 by Phe enhanced the inflammatory response, and it was related to the changes of van der Waals interactions into stronger CH–π interactions with the FA chain, longer than the corresponding one on *E. coli* LPS. The molecular model also showed that Ara4N residues provide additional polar interactions affecting the *B. cenocepacia* LPS binding to the TLR4/MD-2, and contribute to the anchoring of the lipid A into the receptor complex by interactions with both, TLR4 and TLR4*. Interestingly, the presence of the positively-charged ammonium groups in the Ara4N seems to favor the electrostatic interactions and, consequently, the binding, whereas uncharged amino acids are critical for responses to *Bordetella pertussis* lipid A, for example [8]. As described in Section 2.2, this model for the TLR4/MD-2/LPS_BC_ complex was used to generate a computational mutant TLR4/MD-2/LPS_BC_ complex (D294A, R322A, S415A* and S416A*), which was submitted to MD simulations and energy analysis for quantification of the per residue contributions to the final binding energy [7]. Altogether, these results provided a molecular model for the activation of the human TLR4/MD-2 complex by penta-acylated lipid A, which sheds some light onto the comprehension of the molecular recognition of LPS by TLR4/MD-2.

#### 2.3.3. Computational Studies of Non-LPS-Like TLR4 Modulators

**Figure 7:**
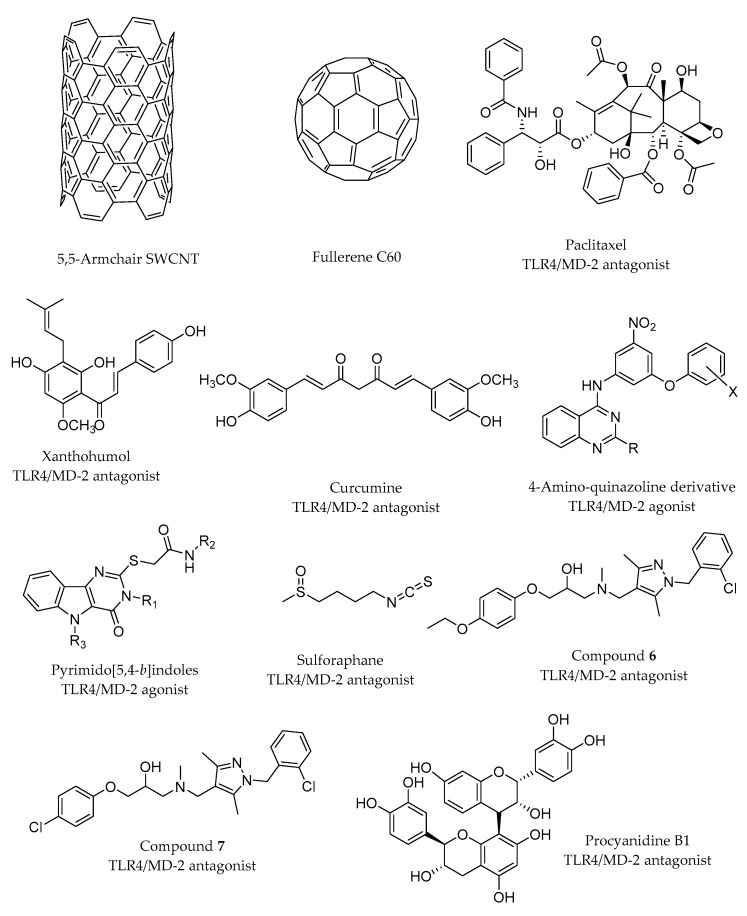
Non-LPS-like TLR4/MD-2 modulators studied by computational approaches.

The species-specific discrimination of TLR4 ligands by MD-2 is exemplified by taxanes, in particular paclitaxel (PTX; Figure 7), a proinflammatory murine TLR4/MD-2 ligand, which activates the subsequent inflammatory cytokine response [40,41,42]. Zimmer et al. demonstrated with different experiments that the activation of TLR4 by PTX requires the *m*MD-2 protein, being independent from TLR4 species [43]. This requirement is due to the electrostatic potential surfaces, hydrophobicity, binding pocket size and the conformational gating of the 123–130 amino acids loop. *h*MD-2 and *m*MD-2 have a very large cavity volume that in principle allows lipid IVa, PTX and Eritoran to fit inside. The study of the electrostatic surfaces of *m*MD-2 (PDB ID 2Z64) and *h*MD-2 (PDB ID 2Z65) by means of SYBYL software [44] shows that the cavities for both structures are close to electroneutral, being *m*MD-2 more electronegative than *h*MD-2, especially in the Cys95–Cys105 loop, which is critical for the MD-2/TLR4 interaction. Furthermore, the electrostatic surface of *h*MD-2 displays three electropositive patches corresponding to Lys58, Lys122 and Lys125, which are absent on the *m*MD-2 surface. Docking studies were performed with the help of the Glide program [32] by using the crystal structure of *h*MD-2 (PDB ID 2Z65) and *m*MD-2 (PDB ID 2Z64). In the best predicted MD-2/PTX binding poses, the benzamido group of PTX is very close to Phe126, suggesting that a π-stacking interaction may exist between both aromatic groups. In addition, the Lys125 side chain establishes hydrophobic contact with the phenyl ring. Another key interaction (cation-π) is established between the phenyl group of PTX and the Lys122, which is the only different amino acid in the MD-2 species-conserved sequence Phe119–Gly123. In MD-2, the multiple interactions attract the Gly123–Lys130 loop so as to form a concave surface facing the docked PTX. The same loop in the mouse protein is oriented in the reverse direction. The presence of a Glu122 instead of the Lys122 in *m*MD-2 leads to a completely different binding pose, possibly due to the absence of the cation-π interaction [43]. Other work by Resman et al. [42] proposed a similar binding mode for paclitaxel and the analogue docetaxel, on the basis of docking performed with AutoDock in *h*MD-2 (PDB ID 2E59). Also in this case, the most favorable docked binding poses of both taxanes oriented the benzoyl group towards the nearby region formed by Ile61, Phe76, Leu78, Phe119 and Phe151 of *h*MD-2.

A docked binding mode for a prenylated chalcone-type (xanthohumol; Figure 7) into the antagonist conformation of *h*MD-2 (PDB ID 2E59) has been proposed by Fu et al. [45]. For this purpose, the Glide docking program was used, and the results highlighted the importance of the H-bonds between the OH groups present in the xanthohumol and residues Tyr102 and Arg90. Moreover, another H-bond between the OH of the phenolic group and Glu92 was identified from the docking studies, but this interaction was rapidly broken during the subsequent MD simulation (50 ns), leading to a final MD-2/xanthohumol complex stabilized by the above-mentioned interactions. An analogue behavior was found for curcumin (Figure 7) after docking with AutoDock also in the same crystal structure of *h*MD-2 (PDB ID 2E59). The *h*MD-2/curcumin complex resulting from the docking was subjected to MD simulations leading to a stable complex with equivalent interactions with Tyr102 and Arg90. Accordingly, experimental studies with MD-2 mutants (MD-2R90A/Y102A) have pointed to a direct binding of curcumin to MD-2 in the same binding site as LPS. This ligand would occupy a large part of the hydrophobic pocket and form H-bonds with residues Arg90 and Tyr102, which were stable along the simulation trajectory. Analogously, the H-bond with Gly92 was broken during the simulation. In addition, MD simulations have revealed that the presence of the ligand stabilizes the complex. In particular, MD simulations of the apo-state and bound state of MD-2 have shown that, in the case of the apo-state, MD-2 suffers an important conformational change, reducing the volume of the cavity entrance, in agreement with other similar MD simulations performed on the apo MD-2 [19,28], whereas the bound MD-2 shows good stability [46].

Cell-based high throughput screening (HTS) allowed the identification of novel chemical entities as potent NFκB activators as selective TLR4 ligands: substituted pyramid[5-4-*b*]indole derivatives [47] and 4-amino-quinazolines [48]. From the former family, one hit compound was selected (Figure 7; R_1_ = phenyl, R_2_ = cyclohexyl, R_3_ = H). A series of pyrimido[5,4-*b*]indole rings with carboxamides substituted with various alkyl, cycloalkyl, aromatic and heteroaromatic groups was synthesized and biologically tested in order to establish the SAR. One of the most active compounds (Figure 7; R_1_ = phenyl, R_2_ = 3,3-dimethylbutyl, R_3_ = H) was docked in the mouse TLR4/MD-2 system. The ligand was predicted to bind within the LPS-binding pocket forming H-bonds with residues Glu439(TLR4) and Arg90(MD-2), and multiple hydrophobic interactions (mainly with the side chains of Leu87, Phe126, Ile124, Phe121, and Phe119 from MD-2). This computational study supported that active compounds appeared to bind primarily to MD-2 in the TLR4/MD-2 complex.

From the second HTS, one 4-amino-quinazoline (Figure 7; R = COOEt, X = H) was identified with selective agonist activity for human TLR4/MD-2 rather than mouse [48]. The docking calculations of this 4-amino-quinazoline into TLR4/MD-2 showed that the ligand establishes hydrophobic interactions with Phe119-121-126 and Leu87 and makes H-bonds with the residues Gln436 and Glu439 of TLR4 and Arg90 of MD-2. Noteworthy is the interaction of the two polar nitro oxygens of the compound with the backbone nitrogens of Ile124 and Lys122 of the MD-2 protein. Moreover, the results from the computational study underlined the importance of the Lys122, which happens to be a glutamic acid in mouse. This could produce an electrostatic repulsion effect with the nitro group, thus justifying the decreased activity in *m*TLR4/MD-2 [48]. Several analogues were synthesized to establish the basis for SAR, confirming the relevant role of the nitro group for the TLR binding and guiding further optimization of the lead compound.

It was shown by liquid chromatography-mass spectrometry analysis that the compound termed sulforaphane (SFN; Figure 7) forms a covalent bond with the residue Cys133 of *h*MD-2. Covalent docking methods were applied in an attempt to explain the propensity of SFN to impair LPS engagement with the MD-2 hydrophobic pocket. The authors proposed a model in which SFN, once covalently linked to Cys133, occupies the same position as the R3” lipid chain of LPS (cf. PDB ID 3FXI; Figure 3b) and XA2 lipid chain of lipid IVa (cf. PDB ID 2E59). More precisely, in their model, SFN is found in close proximity with residues Ile46, Phe76, Phe147, Phe151, Val135 and Leu149 of MD-2. This model suggests that SFN sterically prevents other LPS/lipid A from approaching or settling inside the pocket [49]. The same mechanism was reported for the caffeic acid phenethyl ester compound, using only experimental methods (thus, not reviewed herein) [50].

A series of compounds built by functionalizing pyrazole rings was reported by Bevan et al. [51] to inhibit TLR4 activation. Experimental studies indicated that two compounds (Compounds **6** and **7**; Figure 7) were the lead inhibitors. Thus, these compounds were used for docking studies against TLR4 (using the 3D coordinates extracted from the PDB ID 2Z65). The results indicate that both compounds independently bind at the surface of TLR4 where a protruding loop of MD-2 is normally found in the crystal structure. These predicted binding modes suggest that these compounds compete with MD-2 for binding TLR4, thus preventing or impairing the formation of the TLR4/MD-2 complex, resulting in a TLR4 able to carry out its innate immunity role.

Polyphenol procyanidin B1 (Figure 7) has been shown to be able to regulate innate and adaptive immunity by, inter alia, impairing LPS-induced inflammatory responses in human monocytes [52,53,54]. In order to explain its mode of action at atomic level, the authors undertook experimental and docking studies [55]. They noted a high degree of similarity in terms of the interactions found in the predicted binding pose with the TLR4/MD-2 system when compared to the interactions established by LPS with TLR4/MD-2 in the crystal structure (PDB ID 3FXI). For instance, the phosphate group of LPS forms a hydrogen bond with Ser118 of MD-2 where procyanidin B1 is predicted to form a hydrogen bond with Ser120, which is in close proximity to Ser118. In turn, the binding mode proposed by the authors would suggest that procyanidin B1 impairs TLR4 signaling by successfully competing with LPS to bind to MD-2 inside the hydrophobic pocket.

#### 2.3.4. Computational Studies of Proteins as TLR4 Modulators

Peptide-related molecules have also been explored as putative TLR4 modulators by computational strategies aiming to shed light onto their mechanisms of interaction. Among them, S100A8 is a small protein expressed in neutrophils and platelets, among other cells, and it can be recognized by TLR4, as part of damage-associated molecular patterns [56], thus activating TLR4-mediated immune response. To study how TLR4 recognizes S100A8, a rigid body docking was performed [57]. Human S100A8 crystal structure (PDB ID 1MR8) was docked on mouse TLR4/MD-2 crystal structure (PDB ID 2Z64) using ZDOCK [58], followed by a clustering/re-ranking method. Fifty-four thousand structures were initially generated and ranked, taking into account desolvation and electrostatic energy and shape complementarity. The top five models were examined as possible complex structures. In all of these models, C-terminal residues of S100A8 are located on the interface with TLR4/MD-2, in agreement with experimental data, suggesting that the *C*-terminal region plays a crucial role in TLR4/MD-2/S100A8 recognition.

Another protein, annexin A2 (AnxA2), has been demonstrated to activate human macrophages through TLR4-mediated signaling. Annexins are calcium-dependent proteins that are involved in cell motility, endocytosis and ion channel formation, among others cellular processes [59]. Recently, experimental data suggested that AnxA2 binds to the TRIF/TRAM/TLR4 internalized complex, although the mechanism remains unclear. Protein-protein docking showed how this complex is formed [60]. Since there is no crystal structure available for neither TRIF, nor the TRAM protein, 3D structures were constructed with the SWISS-MODEL homology modeling server [61]. The TRIF model was docked on TRAM using ZDOCK program. The best predicted TRIF/TRAF complex was then docked on mouse TLR4 (PDB ID 3VQ1), and finally, human AnxA2 (PDB ID 4HRE) was docked on this complex. The results showed that the complex is formed through both electrostatic and hydrophobic interactions. Hydrophobic interactions take place between AnxA2 and TLR4 mainly, while AnxA2 establishes strong electrostatic and H-bond interactions with both TLR4 and TRAM proteins. Glu445 of TLR4 interacts with Lys329 of AnxA2, and Glu209, Glu72, Glu73 and Arg223 of TRAM interact with Arg37, Lys302, Arg304 and Asp338 of AnxA2.

A similar approach was used with another protein, the surfactant protein A (SP-A) [62]. This protein downregulates inflammation, binds to TLR4 and stops cytokine release. A protein-protein docking of SP-A trimer on the TLR4/MD-2 complex was performed using GRAMM-X [63]. Among the 100 poses predicted by the server and taking into account experimental data suggesting that SP-A mainly binds to MD-2, only three poses were kept. To identify the interacting residues, binding hotspots were predicted using shape specificity and biochemical contact features. Twelve residues of SP-A were found to interact with the TLR4/MD-2 complex. Using this information, a 20-residue peptide (SPA4) containing the interacting residues of SP-A was synthesized, and it was showed to bind to TLR4 and suppress an inflammatory response.

#### 2.3.5. Computational Studies of Fullerenes, Nanotubes and Dendrimers as TLR4 Ligands

Large molecules have also been subjected to computational studies to unveil their mechanism of action as TLR4/MD-2 binders. Among them, we found interesting examples, such as fullerenes, carbon nanotubes (CNT) and dendrimers. Several studies have indicated a strong impact of carbon nanostructures on the immune system by inducing pro-inflammatory activity through their recognition as pathogens by the TLRs [64,65]. Turabekova et al. have undertaken a theoretical study to analyze 5,5-armchair SWCNT and C_60_ fullerene (Figure 7) interactions with the available X-ray structures of TLRs homo- and hetero-dimer extracellular domains [66]. The authors have searched possible binding sites able to host such nanostructures by identifying the most favorable pockets in terms of hydrophilicity/hydrophobicity and size. In the case of TLR4, the MD-2 binding pocket was detected as the possible binding site. The nanostructures were docked in the environment of the hydrophobic pocket where it interacts with aromatic residues (Phe and Tyr side chains) through π–π interactions and with aliphatic residues through CH–π and lipophilic interactions (Leu, Ile, Ala, Val and Pro). A pair of Lys residues from the rim were found to be accessible for establishing π-cation bonding.

On the other hand, Barata et al. [67] have shown that partially glycosylated polyamidoamine (PAMAM) dendrimer inhibits TLR4/MD-2/LPS-induced inflammation. Molecular modeling studies indicate that the hydrophilic surface bind to the entrance of MD-2 cavity, forming electrostatic interactions between the carboxylic acid branches from the dendrimer glucosamine and the residues lining at the entrance of the MD-2 pocket. Crucially, dendrimer glucosamine interferes with the electrostatic binding between LPS and polar residues Ser118, Tyr102 and Lys91 of MD-2. It was also determined that the bioactivity was due to their surface properties, such as the electrostatic and polar surface, their flexibility and their density.

### 2.4. Virtual Screening Strategies in the Search of Novel TLR4 Modulators

Virtual screening (VS) approaches constitute a current strategy in drug design for the identification of novel chemical entities with a given binding ability. Also in the field of TLR4 research, VS has been recently reported leading to novel ligands with drug-like properties, trying to overcome the solubility problems associated with LPS mimetics.

**Figure 8:**
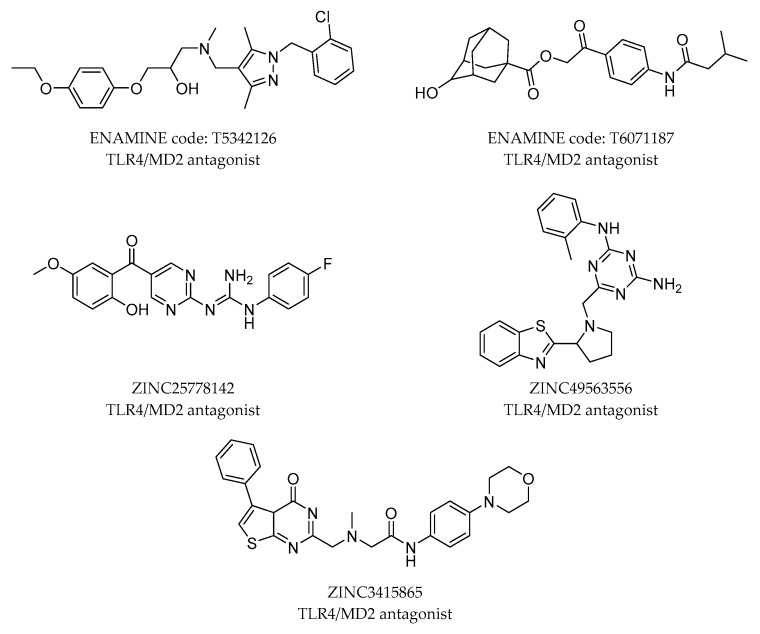
Novel TLR4/MD-2 modulators found by VS approaches.

Among these works, Joce et al. [68] have developed a novel in silico screening methodology incorporating molecular mechanics (MM) and implicit solvent methods to incorporate the evaluation of binding free energies and have screened the Enamine database collection [69]. The resulting clusters were filtered by selecting the representative compounds that were submitted to fast molecular docking for the generation of binding poses and subsequent MD simulations to rank the ligand poses according to their predicted binding affinities. Final filtering led to the identification of compounds T5342126 and T6071187 (Figure 8) as small drug-like inhibitors of the TLR4/MD-2 protein-protein interactions. Their biological activity and selectivity were tested in vitro, and their TLR4/MD-2 antagonist activity was confirmed.

In another study, Švajger et al. [70] performed parallel ligand-based and structure-based virtual screenings in order to identify novel TLR4 antagonists targeting the TLR4/MD-2 interface, by using the ZINC drug-like subset (~11.3 million drug-like compounds) from the ZINC database [71]. The identified ligands after ligand-based VS resulted in being either insoluble in water, or inactive, or presented cytotoxicity on HEK293 cells. However, the structure-based VS identified 40 putative TLR4/MD-2 ligands that were assessed in vitro. After the first assays, only 14 compounds were sufficiently water-soluble and completely non-cytotoxic at 100 μM. These compounds received further biological evaluation, and finally, three compounds with promising antagonistic activities were discovered: ZINC25778142, ZINC49563556 and ZINC3415865 (Figure 8).

## 3. Computational Studies on the Intracellular Domain of TLR4

The intracellular domain of the TLR4 transmembrane protein contains a Toll/interleukin-1 receptor (TIR) homology domain, which is a common feature of all adaptors involved in the initiation of TLR4 signaling, mediating protein-protein interactions between the TLR4 and the signal transduction components. TLR4 has two distinguished signaling pathways involving primarily four TIR-domain-containing adaptors. In the first pathway, the MyD88 adapter-like (Mal) acts as a “sorting” adaptor by recruiting the myeloid differentiation primary response gene 88 (MyD88), the “signaling” adaptor, to the plasma membrane. In the second pathway, the TRIF-related adaptor molecule (TRAM) plays the role of “sorting” adaptor, which recruits the TIR-domain-containing adapter-inducing interferon-β (TRIF), the “signaling” adaptor, to the membrane to initiate the signal. As a major component of theses adaptors, the TIR domain is believed to play a central role in the recruitment processes [72,73].

The crystal structures of human TLR1 (PDB ID 1FYV) and TLR2 (PDB ID 1FYW) revealed the structural basis of the TIR domain [74] followed by the crystal structure of TLR10 TIR domain (PDB ID 2J67) [75] and the solution structure of MyD88 TIR domain resolved by NMR (PDB ID 2JS7 and 2Z5V) [76]. Prior to that release, two homology models of the TIR domain of MyD88 were reported. Both were built based on the TLR2 TIR domain crystal structure (PDB ID 1FYW) resolved by X-ray crystallography [77,78]. In 2012, the crystal structure of Mal was also resolved by X-ray crystallography (PDB ID 3UB2) [79].

**Figure 9:**
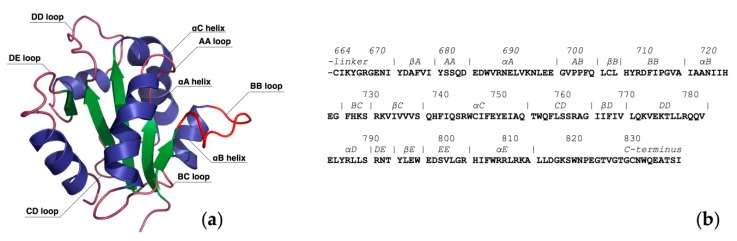
Intracellular TIR domain of TLR4. (**a**) 3D Representation of the homology model; (**b**) FASTA sequence.

The lack of structural information for the TIR domain of TLR4 has driven the creation of models to clarify the recruitment of adaptors from a structural perspective. Dunne et al. [80] built monomer models of TLR4, Mal and MyD88 using comparative modeling and loop refining techniques. They noted differences in the electrostatic surface potentials suggesting that adaptor binding is driven by electrostatic complementarity. This point was also emphasized in a study by Kubarenko et al. [81] in which they compared the surface charges of TIR domains of the crystal structure of *h*TLR2 and of the models of *h*TLR3 and *h*TLR4 and noted that the surface charge distribution of the BB loop and the αC-helix (Figure 9) present similarities in TLR2 and TLR4 and differ between TLR3 and TLR4. The authors considered that these findings could explain why TLR2 and TLR4 recruit MyD88, whereas TLR3 does not. In the computational study by Gong et al. [82], it was highlighted that, whereas the BB-loop is highly conserved among TIR-domains, the APBS electrostatic surfaces differ. The authors hypothesized that this finding might explain the specificity and selectivity of adaptors recruitment. An experimental study showed that a single point mutation in the TIR domain of murine TLR4 (P712H) renders the system hyporesponsive to LPS stimulation. The authors noted that their data do not suggest a direct role for this residue.

**Figure 10:**
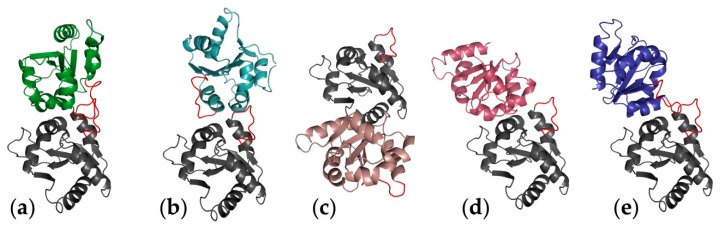
Representation of the different ways the dimer is proposed by published computational strategies to be assembled in the literature by computational strategies. (**a**) First reported by Miguel et al. [83]; (**b**) reported by Gong et al. [82]; (**c**–**e**) reported by Guven-Maiorov et al. [85]. The monomer has been built by homology modeling, and the secondary structure representation has been altered to resemble the other models. The dimers have been assembled manually, fitting as best as possible the schemes present in each paper, to provide an overview of the variety of binding poses reported so far. The dimmers shown do not have the pretention of being as precise as those shown in the original papers and should be considered schematic.

Dunne et al. [80] used a docking procedure based on hydrophobicity and geometry. Their results suggest that Mal and MyD88 bind at two distinct binding sites (non-overlapping): the DD- and DE-loops of Mal forming interactions with the BB-loop and αC helix of TLR4-TIR domain and the AA- and DD-loop of MyD88 with the CD-loop of TLR4 (Figure 9). The biological relevance of this binding mode was later questioned, as it was discovered that TLR4 activation required homodimerization. In line with that, in 2007, Miguel et al. [83] reported the first 3D model of the dimer of the TIR domain of TLR4; a dimer composed of two identical subunits, arranged in a two-fold axis of symmetry (Figure 10a). Despite the observation that some loops are differently oriented, the overall monomeric fold and the secondary structure of each subunit are very similar to the monomer model reviewed above [80]. This dimer model outlines significant interactions between the BB-loops of each monomer; for instance, residues Phe712 are engaged in homotypic aromatic interactions. A flat, but slightly curved surface was observed and attributed to the side facing the membrane. The authors also reported a docking study of TRAM and Mal with the TLR4 dimeric model in which the two adaptors bind at either sides of the dimer interface formed by the union of the two TLR4-TIR domains, which are identical due to the symmetry. They noted that both adaptors are forming strong interactions with TLR4 Trp757. Mal is also interacting with His728, Arg763 and Lys819, whereas TRAM interacts with Glu684, Arg780 and Glu824. The residues of the adaptors found at the TLR4 interface are mostly located on the BB-loop suggesting that the BB-loop of all three TIR-containing structures is of critical importance for binding specificity and selectivity.

Gong et al. [82] performed a docking study based on the geometry, hydrophobicity and electrostatic complementarity of the molecular surface reporting a dimeric model different from the model described above. The interface is formed by residues Pro714 to Ala717 from the BB-loop of one monomer protruding into a groove formed by residues Cys747 to Ile748 from the αC of the other monomer, and vice versa (Figure 10b). In another study, Basith et al. [84], used in silico approaches (homology modeling, protein-protein docking and 5.5-ns MD simulations) to investigate the inhibitory effect of ST2L toward TLR4 activation. ST2L (IL-33r) is a member of the Toll-like/IL-1 receptor superfamily known to negatively regulate MyD88-dependant signaling pathway. The authors reported a TLR4-TLR4 homodimer model [83] (Figure 10a), and their docking study also gave a similar binding mode for Mal (at each side of the dimer). Their results indicate that MyD88 is recruited by Mal, and that ST2L prevents the recruitment of MyD88 by binding at the Mal interface. Thus, according to these results, ST2L successfully competes with MyD88 to bind at the Mal interface.

In a later study, Bovijn et al. [86] reported a homology model constructed based on the crystal structure of the dimeric TLR10 TIR domain. This model is also in agreement with the first model reported by Miguel et al. [83]. The authors proposed that Mal and TRAM adaptors are competing for binding an extended site formed by the reunion of two TLR4 intracellular domains. An experimental mutation study showed that all mutations that impaired Mal binding also impaired TRAM binding, strengthening the idea that Mal and TRAM bind to the same molecular surface. They define the TLR4/TLR4* dimer interface as binding site II, composed of residues from the BB-loop, DD-loop and αC (Figure 9). Then, they describe that the binding site for TRAM and Mal is formed by the reunion of two site I (as defined in the study: residues from αA αB BB and BC), which is in disagreement with the binding site proposed by Miguel et al. [83]. The authors thus argue that their model is supported by experimental data and residue conservation analysis. The binding site III is defined as being located at the opposite direction of binding site I and might be implicated in the interferon regulatory factor 3 (IRF-3) activation.

Singh et al. [87] studied the importance of the highly conserved β-sheets among TLRs’ TIR domain and revealed their primordial implications in the communication network. MD simulations of 100 ns of models based on sequence similarity were performed. MD simulations were used to study the long-range interactions between residues separated by at least 20 residues in the sequence. They reported interactions between the backbone atoms of the first β-sheet with the BB-loop and the third β-sheet. The authors identified four interacting hubs mainly constituted of hydrophobic residues. Among them, three are in the β-sheets just before the BB-loop, the αC helix and the DD-loops, stressing their role in TIR/TIR interaction. This hypothesis was further supported by analyzing the mutations known to completely abrogate signaling. They show that mutants IFI767-769AAA and L815A disturb the interacting network, thus explaining the impaired TIR domain homodimerization capacity. In a very recent paper by Guven-Maiorov et al. [85], the authors used computational techniques to describe the architecture of the signalosome of TLR4. They built three models of the intracellular part of the TLR4 protein (Figure 10c–e). These three dimer models are all unprecedented despite that the secondary structure of the monomer is in great agreement with all of the published models. Furthermore, the authors used two of their models (Figure 10c,d) to propose different binding modes for Mal.

## 4. Conclusions

The relatively recent elucidation of the X-ray crystallographic structure of the extracellular domain of TLR4/MD-2 has opened new perspectives for the research around this challenging receptor. Future development in this field will face the design of non-LPS-like TLR4 modulators, the improvement of the toxicity and drug-like properties, the fine-tuning of the relative potency of the antagonists and agonists and the obtaining of TLR4 agonists-based vaccine adjuvants, among other challenges. Particularly, molecular modeling and computational chemistry techniques have been applied to unravel atomic details about the molecular recognition mechanism of the receptor itself and also about the ligand-receptor interactions of diverse modulators. These findings are herein reviewed and shed light onto their relevance for the future development of novel agonists and antagonists of the TLR4/MD-2 system with promising biomedical applications in sepsis, inflammation, vaccines and cancer immunotherapy, among others.

## Supplementary Materials

Supplementary materials can be accessed at: http://www.mdpi.com/1420-3049/21/8/994/s1.

## Abbreviations

The following abbreviations are used in this manuscript:

Ara4N	4-amino-arabinose
5,5-armchair SWCNT	5,5-single-walled carbon nanotube
AnxA2	Annexin A2
APBS	Adaptive Poisson–Boltzmann solver
CD14	Cluster of differentiation 14
CNT	Carbon nanotubes
CPK	Corey-Pauling-Koltun
FA	Fatty acid
H-bond	Hydrogen bond
HEK293	Human embryonic kidney
*h/m*MD-2	Human/mouse MD-2
*h/m*TLR4	Human/mouse TLR4
HTS	High throughput screening
IRF-3	Interferon regulatory factor 3
LPS	Lipopolysaccharide
Mal	MyD88 adapter-like (also termed TIRAP)
MD Simulations	Molecular dynamics simulations
MD-2	Myeloid differentiation factor 2
MyD88	Myeloid differentiation primary response gene 88
MM-GBSA	Molecular mechanics-generalized Born surface area
MM-ISMSA	Molecular mechanics-implicit solvent model surface area
NMR	Nuclear magnetic resonance
PAMP	Pathogen- associated molecular pattern
PO_4_	Phosphate group
PTX	Paclitaxel
RMSD	Root mean square deviation
RsLA	Rhodobacter sphaeroides lipid A
SAR	Structure-activity relationship
SASA	Solvent accessible surface area
SEAP	Secreted embryonic alkaline phosphatase
SFN	Sulforaphane
TIR	Toll/Interleukininterleukin-1 receptor
TLR	Toll-like receptor
TRAM	TRIF-related adaptor molecule
TRIF	TIR-domain-containing adapter-inducing interferon-β
VS	Virtual screening
WT	Wild-type
