# Supplementary material for: Computational Approaches to Toll-Like Receptor 4 Modulation
Source: Molecules. 2016 Jul 30;21(8):994. doi: 10.3390/molecules21080994 (PMC6274477; doi:10.3390/molecules21080994)
Supplement: Supplementary file 1 [file molecules-21-00994-s001.pdf]

Supporting Materials: Computational Approaches to Toll-Like Receptor 4 Modulation

Jean-Marc Billod, Alessandra Lacetera, Joan Guzmán-Caldentey, and Sonsoles Martín-Santamaría

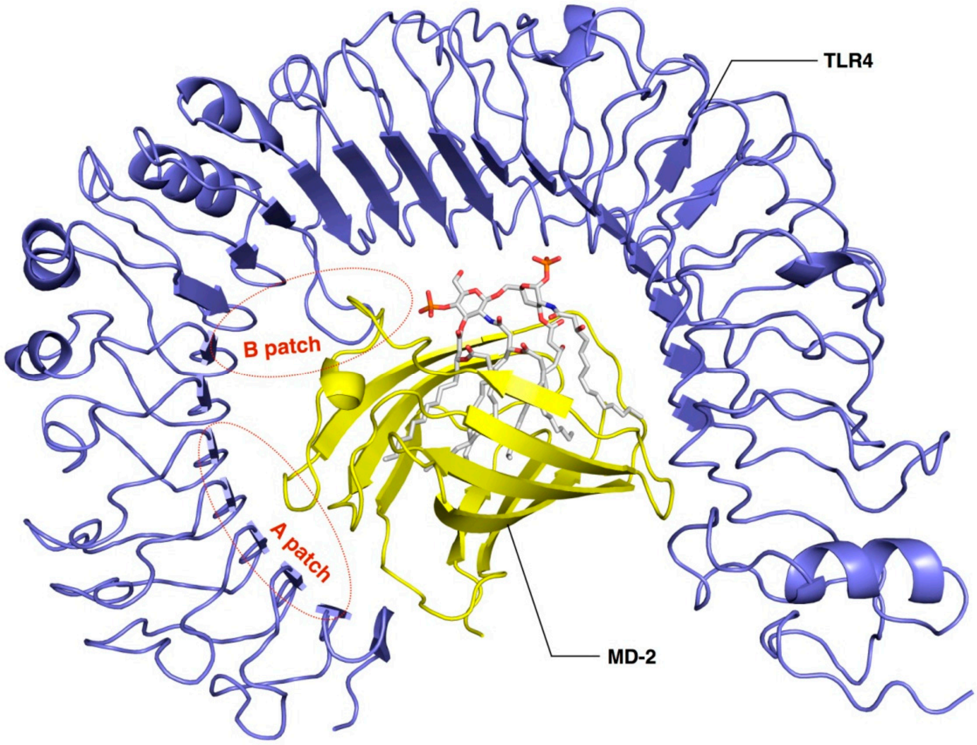

Figure S1. Detailed representation of the TLR4/MD-2 complex from the X-ray crystallographic structure for the extracellular domain (PDB ID 3FXI) in complex with E. coli LPS. Only the lipid A is shown for the sake of clarity. The TLR4 extracellular domain is shown in purple, MD-2 in yellow, and lipid A in green. Protein-protein interacting regions are highlighted: patch A (at the N-terminal domain of TLR4) and patch B (central domain of TLR4).

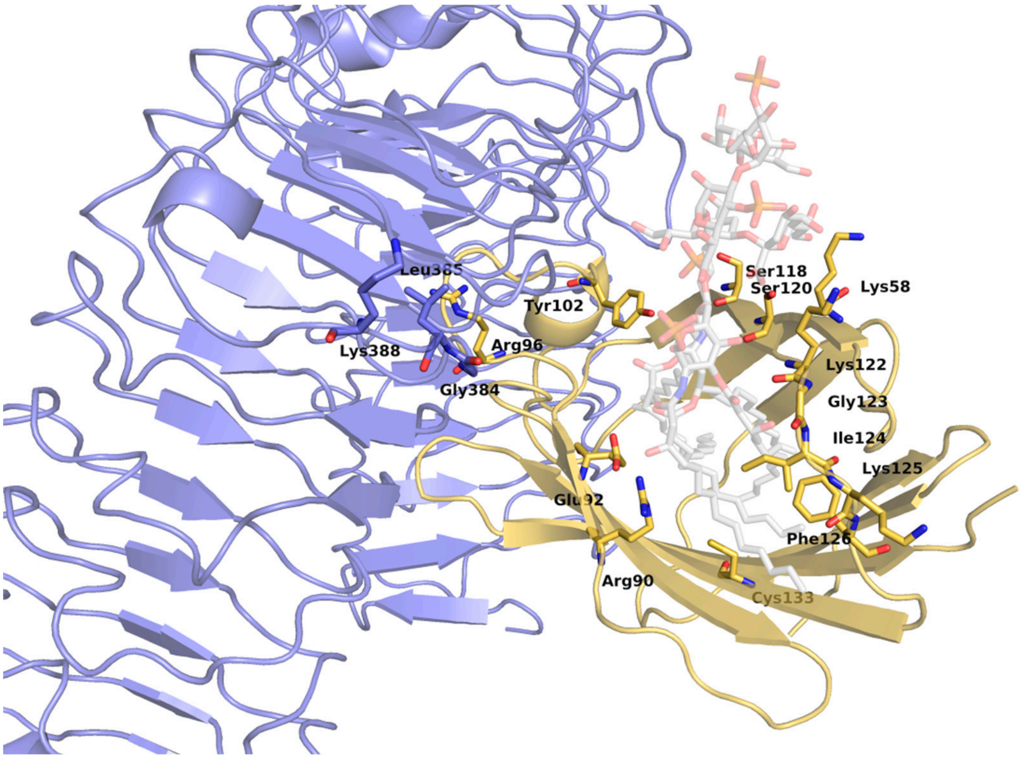

Figure S2. Detailed representation of the TLR4/MD-2 complex from the X-ray crystallographic structure for the extracellular domain (PDB ID 3FXI) in complex with E. coli LPS (CPK colors, partially shaded). Relevant residues discussed in this review are displayed. The TLR4 extracellular domain is shown in purple, MD-2 in yellow.

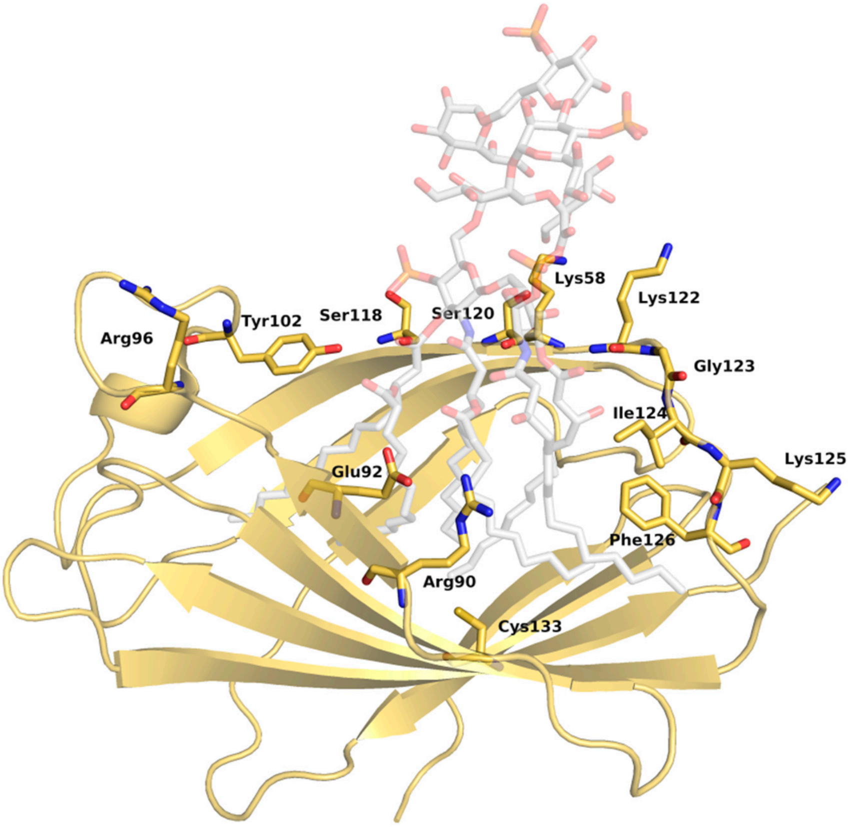

Figure S3. Detailed representation of the MD-2 complex from the X-ray crystallographic structure for the TLR4/MD-2 extracellular domain (PDB ID 3FXI) in complex with E. coli LPS (CPK colors, partially shaded). Relevant residues discussed in this review are displayed. MD-2 is shown in yellow. TLR4 is not displayed for the sake of clarity.
